# Nutritional support and prophylaxis of azithromycin for pregnant women to improve birth outcomes in peri-urban slums of Karachi, Pakistan—a protocol of multi-arm assessor-blinded randomized controlled trial (Mumta PW trial)

**DOI:** 10.1186/s13063-021-05960-9

**Published:** 2022-01-03

**Authors:** Ameer Muhammad, Zoha Zahid Fazal, Benazir Baloch, Imran Nisar, Fyezah Jehan, Yasir Shafiq

**Affiliations:** 1 Present Address: VITAL Pakistan Trust, Karachi, Pakistan; 2 Present Address: Medical College, Aga Khan University, Karachi, Pakistan; 3 Present Address: Department of Pediatrics and Child Health, Aga Khan University, Karachi, Pakistan

## Abstract

**Background:**

Maternal undernutrition is critical in the etiology of poor perinatal outcomes and accounts for 20% of small-for-gestational-age (SGA) births. High levels of food insecurity, antenatal undernourishment, and childhood undernutrition necessitate the supplementation of fortified balanced energy protein (BEP) during pregnancy in low-income settings especially with scarce literature available in this subject. Hence, this paper extensively covers the protocol of such a trial conducted in an urban slum of Karachi, Pakistan.

**Methods:**

The trial is community-based, open-labelled, four-arm, and randomized controlled that will include parallel group assignments with a 1:1:1:1 allocation ratio in low-income squatter settlements in urban Karachi, Pakistan. All pregnant women (PW), if identified between > 8 and < 19 weeks of gestation based on ultrasound, will be offered routine antenatal care (ANC) counseling and voluntary participation in the trial after written informed consent. A total number of 1836 PW will be enrolled with informed consent and randomly allocated to one of the four arms receiving: (1) ANC counseling only (control group), (2) ANC counseling plus BEP supplement (intervention arm 1), (3) ANC counseling plus BEP supplement plus 2 doses azithromycin (intervention arm 2), or (4) ANC counseling plus BEP supplement plus daily single dose of nicotinamide and choline (intervention arm 3).

**Trial registration:**

ClinicalTrials.govNCT04012177. Registered on July 9, 2019.

## Background

Suboptimal nutrition during pregnancy may compromise maternal health, birth outcomes, and offspring development [1, 2]. Undernourishment in utero can stress the developing fetus and adversely reprogram its evolving phenotype [3]. Growth-restricted fetuses are thereafter predisposed to several perinatal health risks [4] including intrauterine growth retardation (IUGR), preterm delivery [5], low birth weight (LBW), immunocompromised status, childhood stunting, and neurocognitive deficits in addition to fetal, maternal, and infant mortality [6–8]. Hence, maternal undernutrition poses a significant global health burden [9], accounting for 7% morbidity [10] plus 70% neonatal [11] and 20% maternal [10] mortality rates worldwide. This situation is even more dismal for resource-constrained and food-insecure countries like Pakistan, where 18% of married women of reproductive age are nutrient-deficient, and thus 44% of children have stunted growth [12].

Maternal undernutrition is, in fact, a significant determinant of LBW in developing countries [13]. An indicator to measure LBW is small-for-gestational-age (SGA), defined as birth weight below the 10th percentile of optimal weight for a given gestational age and sex of a population subset [14]. Of the 23.3 million SGA births globally [15], around 20% are attributable to maternal undernutrition during pregnancy [16]. To address this, the World Health Organization Antenatal Care (WHO-ANC) guidelines recommend the use of fortified balanced energy protein (BEP) supplements during pregnancy to reduce the risk of stillbirth and SGA birth [17]. Following this recommendation, compositional guidance for a ready-to-use supplementary food (RUSF) for pregnant women was developed by the Bill and Melinda Gates Foundation (BMGF) in 2016 [18].

Additionally, the use of prophylactic antibiotics and micronutrients along with BEP supplements have shown to improve perinatal outcomes when used solitarily in various maternal nutritional interventions across low- and middle-income populations [19–21]. For instance, the incidence of preterm delivery and LBW is noted to reduce when two azithromycin doses are administered as a preventive measure for maternal reproductive tract infections [22]. Similarly, a higher maternal choline intake, an essential micronutrient for neurogenesis [23], reportedly improves fetal neurocognitive development [24]. Lastly, dietary supplementation with vitamin B3 or nicotinamide may protect against retarded linear growth in children, as suggested by a recent cohort study [25].

Although substantial data on solitary administration of macro- and micro-nutritional supplements and antimicrobial prophylaxis during pregnancy exists, there is a need for further exploration of the efficacy and synergistic role of these micro- and macro-ingredients on pregnant women (PW) when administered concomitantly. In the context of Pakistan, recent experience with such ready-to-use supplements has been limited. Also, in many cases, these products’ acceptability, efficacy, and effectiveness have not been adequately studied to understand the circumstantial needs. Our study aims to fulfill this lacuna by evaluating and comparing the efficacy of fortified, balanced energy protein (BEP) supplements in malnourished PW from impoverished settings. This will be coupled with or without two prophylactic doses of oral azithromycin and micronutrient fortification with nicotinamide and choline.

### Objectives

The primary objective is to compare the efficacy of fortified, balanced energy protein (BEP) supplements in pregnant women (intervention arm 1) administered solitarily, or in combination with two prophylactic dose of oral azithromycin at weeks 20 and 28 of pregnancy (intervention arm 2), or in combination with daily oral supplements of nicotinamide and choline (intervention arm 3) with that of standard antenatal care (ANC) versus nutritional counseling alone (control arm) in improving the birth weight assessed less than 72 h of birth. The secondary objective is to compare the impact of the interventions on birth length and other anthropometric measurements of the newborn and mother at birth. Further, blood and stool biomarkers of the subjects will be assessed during pregnancy while cord blood, breast milk, and colostrum will be collected at birth. During extended follow-ups from 0 to 11 months, mother-infant dyad will be assessed for anthropometry, and blood and stool specimens will be collected periodically from the sub-sample of the dyad.

## Methods

### Trial design

This is a multi-arm community-based randomized controlled, open-label, assessor-blinded superiority trial with a treatment allocation ratio of 1:1:1:1. A multi-arm trial was selected to determine the incremental impact of BEP to the pregnant woman, along with two doses of oral azithromycin or oral daily nicotinamide and choline on birth weight. This protocol has been developed in accordance with the guidelines set forth by Standard Protocol Items: Recommendations for Interventional Trials (SPIRIT) [26].

### Study setting

The trial will be conducted at Rehri Goth, an impoverished peri-urban coastal slum located along the Arabian Sea belt in the Malir District of Karachi, Pakistan. This ancient site comprises of Sindhi and Baloch ethnicities and a predominant Islamic religion with high illiteracy rate. With approximately 59,000 residents based on a 2017 census, this site has an annual birth rate of around 1500 per year and a cohort of 22% child-bearing-aged female and 3.6% infant population [27]. There are 25 clinics in the area with only one qualified (MBBS) doctor, one government out-patient health center and no maternity home. The lady health workers serving the site belong to Agha Khan Health Services. Major diseases prevalent are diabetes mellitus, skin disorders, and hepatitis [28]. The main occupation in the village is fishing, which is seasonal and yields low income due to unfavorable weather conditions and law restrictions. Seasonal rainfall results in flooding and increases the risk of enteric infections [29]. In the target population, around 27% of pregnant women are malnourished, while about 54% are anemic and approximately 20% of babies are born with low birth weight, according to unpublished data from the VITAL Pakistan Trust (VPT). Thus, Rehri Goth has been a significant focus of VPT operations in tandem with the Aga Khan University Hospital (AKUH) as its partner organization.

### Study population and eligibility

Pregnant women between 13 and 49 years of age will be enrolled if they fulfill the inclusion and exclusion criteria provided in Table 1. New pregnancies in the catchment area are usually identified during routine surveillance rounds and referred to the ANC clinic of VPT. Women with gestational age between > 8 and < 19 weeks based on ultrasound will be screened for eligibility by the research trial team onsite. For eligible participants, written informed consent will be obtained in the local language before they are enrolled for the trial.

**Table 1:** Inclusion and exclusion criteria

Inclusion	Exclusion
▪ Gestational age of 8 to 19 weeks confirmed by ultrasound ▪ A resident of the area for at least the last six months. ▪ Willing to spend the whole pregnancy duration after enrollment ▪ Singleton and viable fetus on ultrasound ▪ Not working and available for ANC and follow-up visits at home ▪ Previously not enrolled in pregnant and lactating women trials	▪ Pregnant women with mid-upper arm circumference (MUAC) ≥ 30.5 cm ▪ Known food allergies

### Sample size

The sample size is calculated via permutation and subsequent adjustment for three comparisons between arms, i.e., intervention arms with control. Improved perinatal outcomes are hypothesized with the supplement of BEP alone or co-administration of BEP supplement with azithromycin as well as with nicotinamide and choline. Thus, the sample size estimated per arm is based on 1-tailed hypothesis testing of birth weight as the primary outcome, a significance level of 2.5% to account for multiple comparisons, and initial assumption of dropout proportion of 21% of study participants assumed in the trial. For birth weight (the primary outcome), it is assumed that the mean difference will be at least 100 g [30]. In order to achieve a test power of 80% and maintain family-wise error rate (FWER) of 0.05; Bonferroni method for three comparisons, alpha of 0.017, and an estimated sample size of 370 pregnant women per arm was attained. A 21% dropout rate was thereafter considered on account of miscarriages, stillbirths, early neonatal deaths, losses to follow-ups, and missed birth assessments due to varying on-field challenges owing to the pandemic. Hence, the sample size was thereafter adjusted to 471 pregnant women in each arm making up a total of 1884 pregnant women for this trial.

### Recruitment

Because married women are included in the surveillance system established by the Department of Pediatrics and Child Health at the Aga University, VITAL Pakistan Trust has access to a list of all the pregnant women in the catchment area. Using that list, all households with pregnant women will be visited by the research team to offer standard ANC services. Thereby, during each pregnancy milestone, antenatal and nutritional counseling will be provided about optimal care-seeking approaches. At the time of clinical ANC visit for delivery, these women will be reapproached by the randomization/enrollment team so that their eligibility is assessed for the trial.

### Informed consent procedure

Eligible pregnant women fulfilling the predefined inclusion criteria will be explicitly explained the aims of the study, procedures and duration of the enrollment. In a household where decision-makers are either husbands or other senior family members (not a woman independently), permission from those decision-makers will be sought to avoid loss to follow-up at a later stage. Adequate time will be given to each participant, i.e., 24–48 h, for discussion with the family members to avoid dropouts and non-compliance. Consent for study participation will be obtained by the research assistant in the presence of a community health worker. To ensure comprehension of the trial and study procedures, clear verbal communication in the study participant’s native language will be imparted. Uneducated participants will be asked to give a thumb impression on the consent form; literate guardians will be requested to sign the consent form.

### Randomization and allocation concealment

**Fig. 1:**
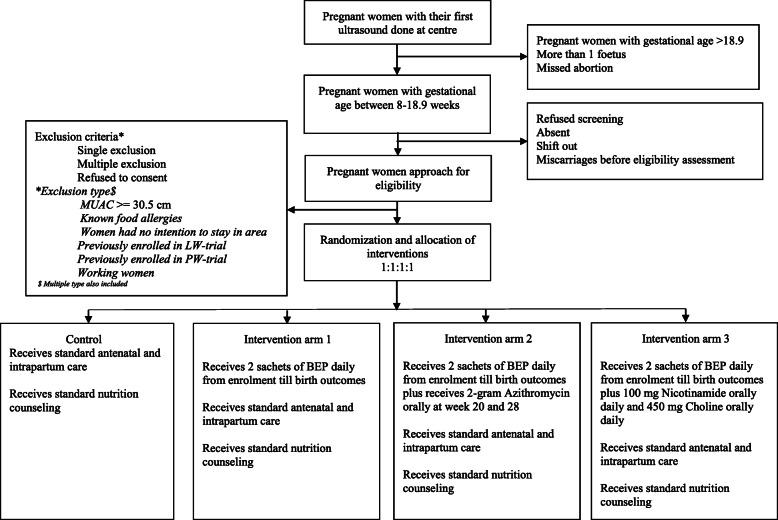
Consort diagram

After written informed consent is obtained, randomization will be performed by the team. Stratified block randomization with blocks of sizes 4, 8, and 12 will be used. Sequence generation will be performed by an independent statistician using a random selection method before the beginning of the trial. Self-adhesive, pre-coded sticky labels with unique identification numbers will be applied to sealed opaque envelopes containing the coded randomization identification number and intervention name to ensure that the randomization process and allocation are blinded. Baseline information regarding nutrition and exclusive breastfeeding will be recorded. Anthropometric measurements of both the mother and newborn will be performed, and follow-up procedures will be explained. Figure 1 shows the trial processes in detail.

### Blinding

The outcome assessors, responsible only for the anthropometry measurements, will be blinded and assigned a schedule that does not overlap with those of the follow-up teams. All investigators will also be blinded to group allocation throughout the period of the study. Moreover, an interim analysis on the blinded arms will be executed by an independent statistician for the Data Safety and Monitoring Board (DSMB). Furthermore, the data analyst performing the final analysis will also be blinded, and the code will eventually be revealed after the blinded results will be shared with DSMB and investigators in a final review meeting.

### Interventions

In the control arm, standard of care, i.e., ANC care, nutritional counseling, health promotional messages of exclusive breastfeeding, and care for intrapartum and postnatal care will be disseminated to all pregnant women. In “Intervention arm 1,” in addition to the standard of care, 2 sachets of BEP supplements per day from enrolment till birth outcome will be allocated to each woman by a trained research team member at initial and subsequent follow-up visits. BEP is a certified product of the World Food Program and is locally produced by the Ismail Industries in Karachi. The manufacturers have no role in any part of the study. Each sachet contains a caloric value of 400 kcal per 75 g and approximately 10.5 g of protein. The sources of protein are mainly chickpea, peanuts, lentils, legumes, and skimmed milk. In “Intervention arm 2,” the same standard ANC counseling BEP interventional doses will be administered to each participant in addition to 2 prophylactic doses of azithromycin tablets 2 g orally at weeks 20 and 28 of gestation (window period of 7 days). In “Intervention arm 3” lastly, daily single doses of nicotinamide 100 mg orally and choline 450 mg orally will be dispensed to each pregnant woman in addition to the same standard of care plus BEP diet supplements. Provision of all ANC services as well as referral for any complication(s) during pregnancy and intrapartum period will be undertaken for all arms. Provision of all ANC services as well as referral for any complication(s) during pregnancy and intrapartum period will be undertaken for all arms. There will be no alternation to the usual care pathways (including use of any medication) and these will continue for all trial arms. All other non-study treatments, such as medications and supplements, will be recorded at each follow-up in all arms. In case any severe illness or adverse event is reported or observed, the intervention may be paused or stopped for a limited period after the investigators and the Data Safety and Monitoring Board are consulted.

### Data collection and data management

Case report forms (CRFs) are designed to capture details on screening, eligibility, randomization, household demography, pregnant assessment, danger signs, severe adverse events, interventional compliance, 24-h food recall (to estimate usual intake plus diversity on a monthly basis), and anthropometry. The data will be collected by a trained team on electronic tablets, with intrinsic logic checks and skip patterns, and updated on secure servers in real time using digital applications, which are designed and built in-house. Auto-alerts will be used to remind about each participant’s follow-up as per schedule, and the data will be tracked according to key indicators. All the data will be collected in a real-time manner and uploaded on a cloud server, which is password-protected and only accessible to the trial data management team and manager. Participants’ confidentiality will be maintained through a unique ID system, and the participant identification information will not be exposed to anyone outside the trial team. The tablets in use will be password-protected and only accessible to the study team. De-identified data will be used for analysis purposes.

### Follow-ups

Domestic visits will be paid by the follow-up teams to provide counseling for all arms. BEP will also be provided to all intervention arms, and compliance will be measured by logging the number of empty sachets since the last visit. Azithromycin oral dose (intervention arm 2) will be given to pregnant women by the research team with close monitoring of adverse events if reported or observed. Similarly, daily administration of nicotinamide and choline will also be the responsibility of the designated follow-up team (intervention arm 3). Follow-ups will be performed daily for the first 15 days following enrollment, every 72 h in the following 2 weeks, and, then weekly in the next stages of pregnancy till birth outcome. At each visit, counseling will be provided to the participants to reinforce adherence to the BEP supplementation and other protocol-related procedures. In addition, monthly 24-h food recall data will also be collected. After birth outcome assessment, the mother-infant dyad will be followed through 0–11 months of infant’s age on a periodic basis. There is no plan of retention for these participants once their 11-month follow-ups are completed. However, through our existing free-of-cost primary health care facilities, standard-of-care is available to all participants, even after the completion of the trial. For participants who move out of the catchment area, a tracking system has been developed so that they can be followed up at the new location in case it is in close proximity. Table 2 shows the schedule of enrollment, interventions, and assessments.

**Table 2:** Time schedule of enrollment, interventions, and assessments

Timepoints	T_**0**_^**a**^	Follow-ups collecting data on key study variables and indicators					Follow-ups for maternal anthropometry	Week 19	Week 32	Primary study endpoint	Extended follow-up Mother-infant dyad (Secondary study end points)
		Daily visits^b^	48 hourly visits ^c^	72 hourly visits ^d^	Daily visits^e^	Weekly^f^	Week 23, 27, 31, 35, 39 of gestation				
										At birth	1–2 months, 3–4 months, 5–6 months and 12 months
**Enrollment**											
*Eligibility assessment*	×										
*Written informed consent*	×										
*Randomization and allocation*	×										
*Baseline data*	×										
**Intervention**											
*Nutrition counseling and ANC*	×	×	×	×	×	×					
*BEP distribution and compliance assessment* ^*g*^	×	×	×	×	×	×					
*Oral Azithromycin administration at week 20 and 28 with daily follow-ups for SAEs monitoring after dose administration* ^*h*^					×						
**Follow-ups during pregnancy**											
*24 h breastfeeding recall*			×	×	×	×					
*Infant assessment for danger sign*			×	×	×	×					
*24 h maternal food intake recall* ^*i*^						×					
*Monthly maternal anthropometry measurements performed by separate team (blinded from intervention details)*							×				×
*Maternal depression*							×	×			
*Participant experience and rating different characteristics of BEP product* ^*g*^							×	×			
**Specimens’ collection during pregnancy**											
*Maternal hemoglobin level* ^*j*^	×								×		
*Maternal ferritin level* ^*j*^	×								×		
*Maternal vitamin D level* ^*j*^	×								×		
*Maternal VAM blood collection* ^*j*^	×								×		
*Maternal urine collection for Choline metabolites* ^*k*^	×								×		
*Maternal plasma collection for proteomics* ^*k*^								×	×		
*Maternal plasma collection for Niacin metabolites* ^*k*^								×	×		
*Maternal stool collection*											
**Birth outcome assessment**											
*Newborn and maternal anthropometry measurements within 72 h of birth performed by separate team (blinded from intervention details)*										×	
**Specimens’ collection soon after birth**											
*Cord blood collection within 30 min of birth* ^*k*^										×	
*Breast milk collection within 72 h of birth* ^*k*^										×	
**Specimens’ collection during extended follow-up**											
*Mother-infant dyad VAM blood collection* ^*k*^											×
*Mother-infant dyad stool collection* ^*k*^											×
**Infant’s growth and neurodevelopment assessment**											
*Monthly infant and maternal anthropometry measurements performed by separate team (blinded from intervention details)*											×
*Neurodevelopment assessment using Mullen, HINE and GSED – at 6 and 12 months of infant’s age plus maternal depression* ^*l*^											×
*MRI scan of infants* ^*k*^											

^a^Window period for eligibility assessment and enrollment is 8–18.9 weeks of gestation

^b^Initial 1–13 days from enrollment

^c^15–25 days from enrollment

^d^29–38 days from enrollment

^e^Pre- and post-Azithromycin administration, i.e., at weeks 20 and 28 of gestation

^f^48 days from enrollment

^g^Only in intervention arm

^h^Only in intervention arm C and window period of 7 days

^i^Only monthly basis

^j^All participants who agreed

^k^Sub-sample of 50 in each arm

### Anthropometry

The teams will be trained to perform anthropometric measurements for each mother-infant dyad using the INTERGROWTH-21st standards by a master trainer who will also conduct monthly refreshers. The measurements will include maternal height, mid-upper arm circumference (MUAC), and weight as well as infant length, weight, MUAC, and head circumference. SECA infant weighing scale model 334 will be used for infantile weight measurement whereas the SECA adult weighing scale model 874 will be used to assess the maternal weight. MUAC tapes from UNICEF will be imported. The SECA scale models 417 and 213 will be used to measure the maternal height and infantile length respectively. The team members recording anthropometric measurements will be blinded to the allocated arms of participants. Data will be entered digitally in the system which will automatically calculate the average value. The allowable difference between the two study measurements according to the standard procedure is ± 0.5 cm for maternal MUAC, ± 0.2 kg for maternal weight, ± 0.5 cm for maternal height, ± 20 g for infant weight, and ± 0.4 cm for infant length, infant MUAC, and infant head circumference.

### Primary outcome

The primary outcome of interest will be the birth weight (g) of the newborn assessed within 72 h of birth.

### Secondary outcomes

The other outcomes of interest will be birth length (cm), head circumference (cm), and MUAC (cm) of the newborn assessed in less than 72 h of birth. For extended follow-ups on days 3, 6, 21, 27, 42, 59, 89, 114, 143, 179, 269, and 359 from 0 to 11 months after birth, anthropometry of the mother-infant dyad will be assessed. Weight velocity (g/day), length velocity (cm/month), length-for-age *z*-score (LAZ), weight-for-age *z*-score (WAZ), and weight-for-length *z*-score (WLZ) will thereafter be calculated. Weight velocity is defined as the incremental change in weight (in grams) per day from birth to 11 months of age. Similarly, length velocity is defined as the progressive change in length (in centimeters) per month measured from birth to 11 months of infantile age. Moreover, the mean differences in the specific *z*-score indicators (LAZ, WAZ, and WLZ) measured at birth and 11 months of age will be assessed. Because anthropometric data for each mother-infant dyad is available on different periodic days as per their follow-up schedule, the aforementioned outcomes assessed according to each follow-up’s duration. Furthermore, the anthropometric measurements of the mother will also be assessed on a monthly basis during pregnancy, i.e., height (cm) (only at enrollment), weight (kg), and MUAC (cm), and body mass index (BMI). The mean change in each of these indicators for different arms will be assessed during pregnancy and in extended postnatal follow-ups.

### Other secondary outcomes

#### Neurodevelopment assessment of infants

In order to assess the impact of maternal interventions on early childhood development (ECD) as growth trajectory, four different approaches or tools will be used to determine the changes at 6 and 12 months of age. This will be undertaken by a team of psychologist and trained senior research staff.

##### Early learning and motor ability

The “Mullen Scales of Early Learning” will serve as a compass for assessing cognitive and motor ability of each infant. The scale will use gross motor, visual reception, fine motor, expressive language, and receptive language to score target strengths and weaknesses among the infants, with each module having specific skip patterns to follow according to age group. The module for infants less than 12 months of age usually takes 25–35 min to complete. This examination will be performed on all eligible infants.

##### Neurological examination

The “Hammersmith Infant Neurological Examination (HINE)” will be used for the neurological examination of infants. It consists of six subscales (tone, tone patterns, reflexes, spontaneous movements, abnormal neurological signs, and behavior) and a total score. There are 34 items, with a maximum possible score of one per item, the points of which are totaled to provide an overall optimality score, as well as the six subscale scores. This will be performed on all eligible infants.

##### Global Scale for Early Development

The Global Scale for Early Development (GSED) aims to fill the neurological and biopsychosocial gap that may present in infancy and childhood through the development of two internationally standardized and validated measurement instruments for the assessment of ECD in children under 3 years of age at population (short form) and programmatic (long form) levels. The instruments are being developed by a multidisciplinary team led by the World Health Organization. Both GSED instruments are constructed from a common item bank (see box for detailed methodology). The first is a short, caregiver-report instrument intended for population-level measurement to assess and map child development status, draw attention to populations in most need of support, track trajectories of child development over time at a population level, and monitor benefits of national-level policies and programming. The second is a longer instrument for program evaluation that combines direct assessment and caregiver report to quantify the impact of an intervention on early developmental outcomes. Both forms are developed to be culturally neutral, easy to administer; open-access and freely available; acceptable and understandable to caregivers and children; and easily interpretable by policymakers and program personnel. The instruments are designed to be holistic measures for interpretation at the population or group level. They are not intended for individual diagnosis or screening of children. The data collected via GSED will provide the conceptual and empirical basis for the future development of “norms” that can be used to monitor the proportion of children who are developmentally on track [31].

##### Brain morphology

On a sub-sample of 50 infants per arm, brain imaging will be performed using magnetic resonance imaging (MRI) to assess brain volumes and morphometry. The portable low-field scanner, “Hyperfine MRI machine” will be used for this purpose. Approved by the Food and Drug Authority (FDA) [32], this new diagnostic tool will be used to assess brain development in complement to other ECD assessments mentioned above.

#### Participant experience and rating different characteristics of BEP product

At weeks 19 and 32 of gestation, the participants in the intervention arms will be approached to share their product experience with BEP supplements. This will include product taste, odor, texture, physical appearance, and packaging.

#### Maternal depression

##### Antenatal period

Pregnant women will be screened for depression at weeks 19 and 32 of pregnancy by a team of one psychologist and trained senior research using the “Patient Health Questionnaire 9-item tool (PHQ9)” will be used to screen the. In subject(s) requiring urgent care based on the finding, a referral for higher-level care will be furnished for further diagnosis and treatment.

##### Post-partum period

Using Edinburgh Postnatal Depression Scale (EPDS) tool, data on maternal depression at 6 and 12 months post-partum will be collected by the same team of one psychologist and trained senior research staff. In a case where participant(s) require urgent care based on the finding, a referral for higher-level care will be furnished for further diagnosis and treatment.

#### Biomarker assessment during pregnancy period

##### Maternal blood specimen collection

Maternal blood will be collected at enrollment and 32 weeks of pregnancy from all consensual participants to assess the levels and differences of hemoglobin (g/dl), ferritin (ng/ml), and vitamin D (ng/ml) from each arm. Separate blood specimens for metabolomic analyses will be simultaneously drawn using the “Volumetric Absorptive Microsampling” (VAM) technique.

On a sub-sample of 50 pregnant women per arm approached via convenience sampling, plasma for Niacin metabolites and for niacin co-enzymes, specifically erythrocyte nicotinamide adenine dinucleotide (NAD) and nicotinamide adenine dinucleotide phosphate (NADP), will also be collected at the same time point to compare the levels of these metabolites among the four arms with those receiving an extra daily dose. On this same sub-sample, plasma for proteomic analysis will be collected at 19 and 32 weeks of pregnancy to gain an in-depth analysis of proteome and assess the potential impact of administrating azithromycin.

##### Maternal urine specimen collection

Urine sample will be collected from the same sub-sample as above at enrollment and 32 weeks of pregnancy to see how the level of choline metabolites differ in the four arms compared to the one receiving an extra daily dose of choline.

##### Maternal stool specimen collection

Stool specimen on the same sub-sample of 50 pregnant women per arm will also be collected at 19 and 32 weeks of pregnancy. The specimens will be assessed for inflammatory biomarkers in the stool, such as calprotectin (μg/g), lipokalin-2 (pg/ml), and myeloperoxidase (MPO), using ELISA. Furthermore, the stool samples will also be analyzed for enteropathogens using the TaqMan Array Card (TAC) system for polymerase chain reaction (PCR), which will be performed in the Infectious Disease Research Lab (IDRL). Moreover, a targeted Bifidobacterium identification will be performed using real-time PCR at IDRL. The stool samples will be sent to the University of Stanford after signing the material transfer agreement (MTA) for additional metagenomic and microbiome analyses.

#### Biomarker assessment during pregnancy period

##### Cord blood collection

On the group of the same sub-sample as above, cord blood will be collected within 30 min of birth using dried blood spot (DBS) cards to assess micro- and macronutrients and antibody status in the cord blood.

##### Breast milk collection

Colostrum from the same women will be collected within 72 h of birth to assess the quality and composition of breast milk in terms of macro- and micronutrients, including oligosaccharides, immunoglobulins, and microbiome. The analysis of breast milk specimens will be performed in the Azad Lab at the University of Manitoba. A material transfer agreement (MTA) will be developed with the University of Manitoba for the shipment of the specimens.

##### Maternal and infant stool specimen collection

Stool specimen on the same sub-sample of 50 mother-infant dyad per arm will also be collected at 1–2, 3–4, 5–6, and 12 months of infantile age. Specimens will be assessed for inflammatory biomarkers in the stool, such as calprotectin (μg/g), lipokalin-2 (pg/ml), and myeloperoxidase (MPO), using ELISA. Furthermore, the stool samples will also be analyzed for enteropathogens using the TaqMan Array Card (TAC) system for PCR, which will be performed in the Infectious Disease Research Lab (IDRL). Moreover, a targeted Bifidobacterium identification will be undertaken using real-time PCR at IDRL. The stool samples will be sent to the University of Stanford after signing the material transfer agreement (MTA) for additional metagenomic and microbiome analyses.

##### Blood specimen collection

From the same 50 mother-infant dyads per arm, infantile blood specimens will also be simultaneously collected at 1–2, 3–4, 5–6, and 12 months of infantile age for metabolomic work using Volumetric Absorptive Microsampling (VAM). The specimens will be stored in − 80 °C freezers at the IDRL and NRL storage areas of the Aga Khan University for further analysis in future research for an indefinite time. The samples will be color-coded by the type of specimen as part of a unique identification system and de-identified with barcodes, specific IDs for different time points, and mother-infant dyad information. All the ethical aspects pertaining to the storage of these samples have been approved by the Ethics Review Committee at the Aga Khan University.

### Monitoring and quality assurance

A specific team from the Aga Khan University, with expertise in data management and trial implementation working with the investigators, will be responsible for auditing the general data trial processes and training the research staff and outcome assessors to ensure the completeness and accuracy of the protocols. Moreover, the Technical Advisory Group (TAG) comprising of international experts will be evaluating the trial progress and independent experts will visit the catchment periodically for monitoring. A mechanism has also been developed for the research teams to report key progress indicators on a weekly basis to the investigators at the VITAL Pakistan Trust and Aga Khan University. Furthermore, an astringent quality assurance mechanism has been developed through which 10% of randomly selected data will be checked by trial supervisors and associates. The anthropometric measurements will be standardized, and the team members will be trained by WHO-trained master trainers. Comprehensive training and refreshers will be conducted on a routine basis for the research team members, all of whom have received Good Clinical Practice certifications.

### Data safety and monitoring board

The independent group of experts, comprising of 5 members, constitutes the Data Safety and Monitoring Board (DSMB) for the trial and are responsible for monitoring safety indicators, adverse events, and results of the interim analysis. The interim analysis (blinded by arm) is scheduled for when 50% of the enrolled participants complete the 6-month follow-up. Only DSMB members will have access to the results of interim analysis, which will be shared by the independent statistician. Data on severe adverse events will be shared with the board on a monthly basis in the form of a progress report.

### Participant safety

Close follow-up will be performed to ensure participant safety, and pregnant women will be referred to physicians at the primary health care clinic if needed, with facilitated referral to tertiary hospitals when required. An independent DSMB will monitor the safety of the study participants and practice trial oversight. Monthly reports on severe adverse events will be shared with DSMB, and when a safety red signal is observed, DSMB may stop this trial prior to the completion of recruitment. The safety net, including facilitated referral and reporting, is believed to minimize the chances of harm to participants.

### Possible risks

Trial participants may experience diarrhea, nausea, vomiting, skin rash, and abdominal distension after BEP supplementation and/or following azithromycin, nicotinamide, or choline doses. Information of all adverse event occurrences at each follow-up will be collected systematically via exhaustive history-taking for any illness since the past visit and assessing signs of concern at each visit. If the pregnant women are considered to have any concerning sign or symptom, a referral mechanism has been put in place. A 24/7 active contact number will be provided to the participants to report any serious illness, for which immediate referral will be arranged. This information will be well-documented and recorded under the “severe adverse events” field. For reporting to the ethics committee and DSMB, “severe adverse events” has been divided into two main categories: “fatal events” that may be occurring regardless of the underlying cause, and “non-fatal events” that may require hospitalization or injectable therapy during the follow-up period. Risk management includes prevention through rigorous follow-ups, and continuous monitoring, documentation, and prompt referrals for danger signs. Every illness or danger sign reported or identified is addressed through facilitated referral for both women and infants.

### Statistical analysis

All analyses will be done using Stata software version 16. Descriptive analysis of each group will be conducted, and percentages or continuous data with SD will be reported. The baseline characteristics will be assessed by arm. The primary approach will be intention-to-treat (ITT) analysis to compare primary and secondary growth outcomes. Mean differences will be assessed using one-way ANOVA, with the model adjusted for baseline characteristics including maternal age, BMI, gestational age, gravidity, MUAC, and newborn age at birth assessment. Key assumptions are that outcome data will be repeatedly measured, normally distributed, and following the superiority principle. For covariate analysis, both the unadjusted and stepwise-adjusted models will be run. Point estimates less than 10% will be reported as unadjusted despite of the model employed. Potential sub-group analyses for primary and secondary outcomes will be based on infantile gender and maternal data at enrolment, i.e., MUAC < 21.0 cm or ≥ 21.0 cm, age < 30 years or ≥ 30 years, gravidity < 3 or ≥ 3, and BMI < 18.5 kg/m^2^ or ≥ 18.5 kg/m^2^.

### Participants and public involvement

The investigators have extensive experience working with the community and their representatives/elders. During the protocol development phase, the team discussed and received feedback on the research question and trial design from the community representatives. Furthermore, community perspectives about the trial procedures, especially the frequency and duration of follow-up and biospecimen collection procedure, were also considered. Additionally, during the pilot phase, the aim was not only to test the consent form and questionnaires but also to understand the community response to different questions and how sensitive information regarding the antenatal and postnatal periods can be collected in a receptive and profound manner.

### Ancillary care

VPT will pay for ancillary care in case the participants incur any harm owing to trial participation or have health issues not monetarily covered by the national healthcare program.

### Confidentiality

During the trial, the data files containing personal identifying information will be stored in a password-secured server. Only the members in the top hierarchy of the research team, i.e., principal investigator and project coordinators, will have access to those files. Moreover, biological samples collected from the field site will be de-identified to respect the privacy of study participants.

### Dissemination plan

Processes will be developed to facilitate collaborative data-sharing for scientific purposes. De-identified data with analytical/statistical codes will be made available to the public domain 2 years after the publication of the principal manuscripts. However, investigator support will be needed for which the approval of the proposal and counter-signatures of the data access agreement are a requisite. The data will be uploaded on a password-secured cloud server at all times.

## Discussion

This paper describes the protocol of an open-label, randomized controlled trial in the Rerhi Goth slum. The control and intervention groups will both receive standard ANC counseling. Additionally, PW will receive a BEP supplement coupled with prophylactic antibiotics or micronutrients in the intervention group. This study will thus allow us to assess neonatal anthropometric velocity through improved maternal nutrition. The salient features of the present trial are the co-administration with azithromycin or choline and nicotinamide with BEP supplements for comprehensive learning of nutritional supplement efficacy in the study population. The administration of these supplements at particular lapses of pregnancy will enable us to assess the specific titer of prenatal supplementation needed to yield optimal perinatal outcomes. Close surveillance of daily supplementation will ensure compliance and prevent misutilization of the interventional products.

VPT has already preceded several similar trials in the study setting in the past. This promises a better hindsight of the issues that arose in the previous trials and alleviate them for the upcoming studies. For instance, pregnant women who had stable jobs were usually non-compliant to the doses dispensed. This posed problems with supplementation compliance in the past and was considered during the current trial, thus leading to their exclusion from participation in this study. Multiple sub-studies nested in the main trial can provide insight into the mechanism by which prenatal BEP supplementation affects perinatal outcomes. And last, similar studies are being conducted in other developing countries, allowing for comparison of results from different contexts.

The Mumta PW trial will thus cite the impact of BEP supplements on neonatal and infantile anthropometric outcomes using a rigorous study design. The study results will further strengthen and refine the WHO’s recommendation on using context-specific BEP supplementation during pregnancy for impoverished settings.

### Strengths and limitations of this study

The study was uniquely designed from the perspective that robust data on nutritional interventions are grossly lacking for pregnant women in resource-impoverished settings, let alone the impact of such interventions combined with a prophylactic dose of azithromycin or nicotinamide and choline. Potential biases which could have imposed foreseeable limitations on the trial results are enlisted below. The respective countermeasures undertaken to eliminate these biases are also mentioned.

1. Selection bias: This may have occurred due to the unblinded nature of the study and has been mitigated by independent allocation-sequencing and block randomization.
2. Performance bias and detection bias: The blinding of the outcome assessors to the study arms through independent teams will decrease this bias.
3. Incomplete outcome data (attrition bias): The period of follow-up throughout pregnancy and beyond to 12 months of infantile age, is substantially long and leads to a risk of participants being lost to follow-up. However, efforts will be made to mitigate this through communication with the community in the trial run-in period and connecting with families. If a participant moves away from the catchment area, they will be followed at their new address until the outcome of the trial if possible. Regardless, all available data will be used during analysis, even if there is missing data at certain time points.
4. Non-compliance bias: Non-compliance can be caused by the taste and potential adverse effects of the supplements and long duration of consumption. This will be addressed through detailed counseling at the time of consent and sufficient time provision to potential study participants for decision-making and/or seeking the input of the household decision-makers. Continuous counseling at each follow-up throughout all arms is also the key to prevent dropouts.
5. Contamination: This will be prevented by enrolling only one participant from a single household and through the provision of excess supplements, as women are likely to feed the supplements to other members of the family.

### Trial status

Active protocol version number: 1.6, October 9, 2021. Protocol amendments have been submitted, and the details of the protocol versions with the date of the amendment are provided in Table 3. Started on 22nd July 2019, the recruitment is currently ongoing and is expected to be completed in January 2022, while the last follow-up is expected to be completed in December 2023. The database will be locked in June 2024.

**Table 3:** Protocol versions

Version	Date and changes
1.0	May 21, 2018—Original protocol
1.1	June 16, 2018—Proposed dose azithromycin as 1 g at weeks 20 and 28 of gestation
1.2	August 24, 2018—Proposed dose azithromycin as 2 g at weeks 20 and 28 of gestation
1.3	January 26, 2019—Addition of fourth arm
1.4	August 15, 2020—Addition of secondary outcomes like specimens, extended follow-ups, and neurodevelopment assessment
1.5	April 12, 2021—Increase in sample size based on new dropout rates due to C0VID-19 pandemic impact of health services
1.6	October 9, 2021—Recalculation of sample size based on TAG recommendations for multiple arm comparison

## Data Availability

Processes will be developed to facilitate data-sharing for scientific purposes in a collaborative manner. De-identified data with analytical/statistical codes will be available to the public domain for 2 years after the publication of the main manuscripts with investigator support once proposal approval is granted and data access agreement has been signed. The data will be uploaded on a password-secured cloud server.
